# The Intersection of HPV Epidemiology, Genomics and Mechanistic Studies of HPV-Mediated Carcinogenesis

**DOI:** 10.3390/v10020080

**Published:** 2018-02-13

**Authors:** Lisa Mirabello, Megan A. Clarke, Chase W. Nelson, Michael Dean, Nicolas Wentzensen, Meredith Yeager, Michael Cullen, Joseph F. Boland, NCI HPV Workshop, Mark Schiffman, Robert D. Burk

**Affiliations:** 1 Division of Cancer Epidemiology and Genetics (DCEG), National Cancer Institute, National Institutes of Health, Rockville, MD 20850, USA; megan.clarke@nih.gov (M.A.C.); cnelson@amnh.org (C.W.N.); deanm@mail.nih.gov (M.D.); wentzenn@mail.nih.gov (N.W.); yeagerm@mail.nih.gov (M.Y.); michael.cullen@nih.gov (M.C.); bolandj2@mail.nih.gov (J.F.B.); schiffmm@mail.nih.gov (M.S.); 2 Sackler Institute for Comparative Genomics, American Museum of Natural History, New York, NY 10024, USA; 3 Cancer Genomics Research Laboratory, Leidos Biomedical Research, Inc., Frederick, MD 21701, USA; 4 NCI HPV Workshop, DCEG, Rockville, MD 20850, USA; sara.bass2@nih.gov; 5 Departments of Pediatrics, Microbiology and Immunology, Epidemiology and Population Health, and Obstetrics & Gynecology and Women’s Health, Albert Einstein College of Medicine, Bronx, NY 10461, USA

**Keywords:** HPV carcinogenesis, HPV epidemiology, HPV genomics, viral–host interactions, HPV16

## Abstract

Of the ~60 human papillomavirus (HPV) genotypes that infect the cervicovaginal epithelium, only 12–13 “high-risk” types are well-established as causing cervical cancer, with HPV16 accounting for over half of all cases worldwide. While HPV16 is the most important carcinogenic type, variants of HPV16 can differ in their carcinogenicity by 10-fold or more in epidemiologic studies. Strong genotype-phenotype associations embedded in the small 8-kb HPV16 genome motivate molecular studies to understand the underlying molecular mechanisms. Understanding the mechanisms of HPV genomic findings is complicated by the linkage of HPV genome variants. A panel of experts in various disciplines gathered on 21 November 2016 to discuss the interdisciplinary science of HPV oncogenesis. Here, we summarize the discussion of the complexity of the viral–host interaction and highlight important next steps for selected applied basic laboratory studies guided by epidemiological genomic findings.

## 1. Background

High-risk human papillomaviruses (HR-HPVs) cause a heavy burden of cancer with more than 600,000 cancers attributed to HR-HPVs worldwide in 2008 [1,2,3]. Of these patients, 236,000 deaths are estimated to be caused by cervical cancer alone each year [4]. Yet, the genetic basis of HPV oncogenicity, firmly established for one clade of the genus *Alphapapillomavirus* [5], has not been solved. This research question is now tractable, given the strong genotype-phenotype associations, the availability of fundamental molecular biological knowledge regarding the small HPV genome, and recent advances in high-throughput sequencing of HPV genomes. 

Papillomaviruses (PVs) are circular, double-stranded DNA viruses that are believed to have co-diverged with animal and human host populations for millions of years, and are ubiquitous throughout the world [6,7,8,9]. HPV genomes are approximately 8000 base pairs in length and contain eight to nine open reading frames (ORFs) that encode highly conserved core proteins involved in viral genome replication (E1 and E2/E4) and assembly (L1 and L2), as well as accessory proteins (E5, E6, and E7). While E1 and E2 are involved in HPV replication and in regulating viral transcription, the primary oncogenes, E6 and E7, are thought to be largely responsible for niche adaptation, viral amplification, and inadvertently driving carcinogenesis [10]. Niche adaptation refers to the virus adapting to a specific anatomic/cellular region. All HPV genomes contain E7, and a few lack E6 and E5 [11]. The upstream regulatory region (URR) is a non-coding region containing cis-responsive elements that regulate replication and transcription of viral proteins [12]. 

HPV types are classified based on pairwise nucleotide sequence identity within the highly conserved *L1* gene, and distinct types (e.g., HPV16 vs. HPV31) are defined by differences of at least 10% at the nucleotide level [13]. An HPV species group (e.g., *Alphapapillomaviruses-9*) comprises HPV types sharing ≥70% of their L1 sequences [13]. To date, more than 200 HPV types have been identified and characterized [13]. Some HPV types within the alpha-HPVs infect mucosal epithelia and are associated with a variety of outcomes, ranging from benign asymptomatic infections to genital warts and cervical cancer. Of the ~60 alpha-HPV types, 13 from a single clade (i.e., branch of the phylogenetic tree) have been classified as definitely or probably carcinogenic (high-risk) and account for >95% of all cervical cancers worldwide. These include HPV16, HPV31, HPV33, HPV35, HPV52 and HPV58 (*Alphapapillomaviruses-9* species group); HPV18, HPV39, HPV45, HPV59, and HPV68 (*Alphapapillomaviruses-7* species group); HPV51 (*Alphapapillomaviruses-5* species group); and HPV56 (*Alphapapillomaviruses-6* species group) [14]. On a finer scale, within each of these HPV types there are variant lineages and sublineages with intratypic genome sequence differences of 1.0–10% and 0.5–1.0%, respectively [15]. 

**Figure 1:**
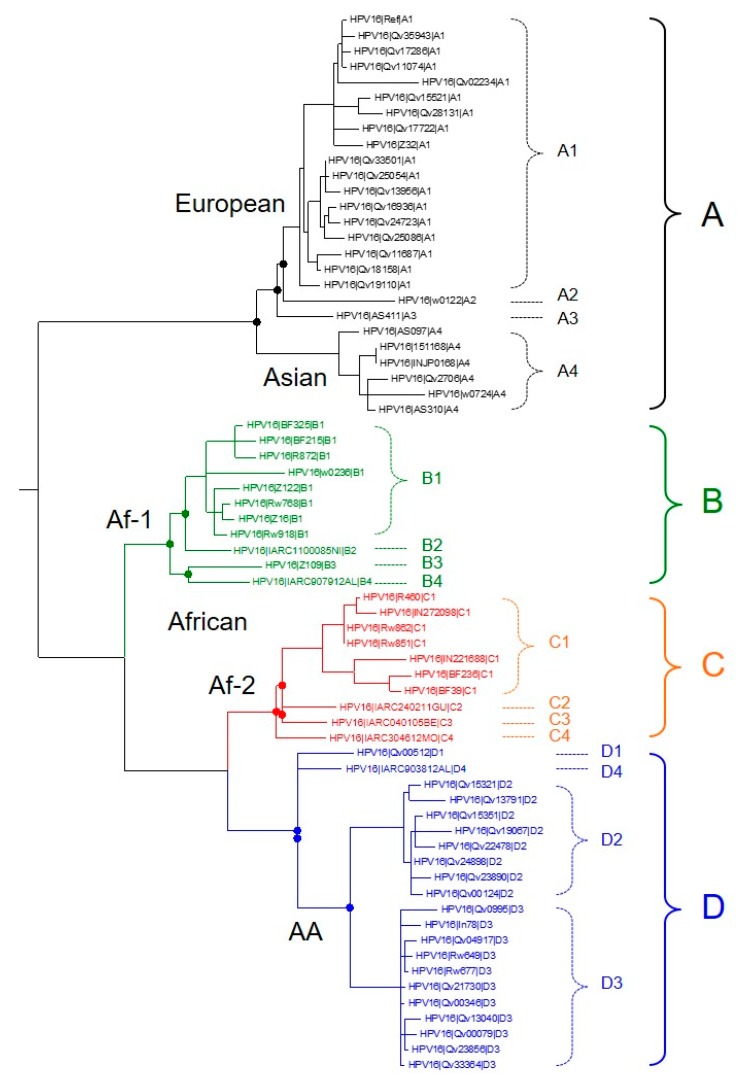
Phylogenetic tree illustrating human papillomavirus 16 (HPV16) lineages (A–D) and sublineage (A1–4, B1–4, C1–4, D1–4) relationships. Colors indicate main lineage branches. A maximum likelihood tree is shown inferred from 66 HPV16 whole-genome sequences, modified from Burk et al., 2013 [15], including additional reference sequence data from Mirabello et al. 2016 [21], 2017 [37]. Af-1, African-1; Af-2, African-2; AA, Asian-American.

HPV16 is by far the most carcinogenic HPV type, associated with approximately 50% of all cervical cancers, the majority of other HPV-related anogenital cancers, and more than 80% of HPV-positive head and neck cancers [16,17,18,19,20]. HPV16 variant lineages have been extensively studied, with four major variant lineages and up to 16 sublineages identified to date (Figure 1), including: sublineages A1-3 (traditionally classified as European), A4 (Asian), B1-4 (African-1), C1-4 (African-2), D1 (North American), D2 and D3 (Asian-American), and D4 [15,21]. Several studies have demonstrated that HPV16 variant lineages and sublineages confer differential risks of persistence, and progression to cervical precancer and cancer [21,22,23,24,25,26,27,28,29,30,31,32,33] (reviewed in Burk et al. [15]). The epidemiologically-defined co-factors, smoking and use of hormonal contraceptives, do not modify HPV16 variant lineage risk substantially [28], however, HPV16 lineages have not been evaluated considering host genetic factors and this could modify risk slightly. Some authors further showed that specific HPV16 lineages are associated with glandular versus squamous histology [21,26,32,34,35,36]. 

Recent advances in high-throughput next-generation sequencing [38] have enabled the large-scale study of HPV genome variability and led to new discoveries in HPV genomic research. These findings based on empirical population-based studies provide opportunities for further investigation at the intersection of molecular biology and epidemiology that could enhance our molecular understanding of HPV-related carcinogenesis. In recognition of this exciting and unique opportunity, the National Cancer Institute’s (NCI) Division of Cancer Epidemiology and Genetics (DCEG) sponsored a workshop entitled “Mechanistic Understanding of Cervical Carcinogenesis” on 21 November 2016. Planning and organization of the workshop was led by Lisa Mirabello and Robert D. Burk of the NCI-DCEG HPV Genomics Group. The primary goal of this workshop was to promote interdisciplinary discussions on the potential mechanisms underlying differences in carcinogenicity at the HPV type, lineage, and nucleotide levels and the potential next steps. The workshop brought together an expert panel spanning biochemistry, molecular biology, evolution, pathology, epidemiology, bioinformatics and statistics (Supplementary Materials Table S1). Some of the members of the NCI-DCEG HPV Genomics Group (Robert D. Burk, Mark Schiffman, Nicolas Wentzensen, and Lisa Mirabello) briefly presented the latest HPV epidemiologic and genomic data, and the majority of the workshop was focused on panel discussions addressing specific questions about the molecular mechanisms of HPV carcinogenesis defined by differences in HPV genomes that have remained unresolved (Table 1). This report summarizes the main highlights from this Workshop, with the goal of stimulating further research to understand the specific mechanisms underlying HPV carcinogenesis.

## 2. Recent Discoveries in HPV Genomics

Through an international collaborative effort, the NCI HPV Genomics Project has sequenced many thousands of HPV genomes from well-characterized populations. With an initial focus on HPV16, recent data have confirmed and expanded earlier reports relating precancer/cancer risk to particular HPV16 lineages and uncovered several remarkably strong associations between HPV16 genetic variation and cervical carcinogenicity, as well as providing new insights into HPV diversity in the population. We have applied lineage-based and agnostic, gene and single nucleotide polymorphism (SNP)-based approaches to studying HPV genetic variation [21,38]. At the lineage-level, HPV16 sublineages confer differing risks of precancer and cancer, and most strikingly, differing risks of glandular lesions [21,26,32,34,35,36] which are of rising public health importance—they are more difficult to detect with cytology, have a poorer prognosis than squamous cell carcinoma (SCC), and their proportion among all cancers have been shown to be increasing in many developed regions [39,40,41,42,43,44]. The HPV16 sublineages, A4, D2, and D3, have significantly increased risks of adenocarcinoma in situ (AIS) and adenocarcinoma (ADC) compared to the most common A1/A2 sublineages; D3 and D2 have the strongest risks of ADC with relative risks of 59 and 137, respectively [21]. This indicates that only a small number of genetic differences (e.g., D and A HPV16 sublineages differ by <2.0% of 7906 nt) lead to large differences in risk of ADC. 

Next-generation sequencing (NGS) HPV genome data with deep sequence coverage allows the sensitive identification of within HPV16 variant lineage co-infections. Using these data, within type co-infections are suspected in women with multiple “heterozygous” allele calls (HPV is a monoploid genome). The co-infections can be confirmed by the identification and visualization of multiple lineage-specific sequence variants occurring in shared sequence reads, representing two separate HPV16 variant lineage isolates. In the case of multiple lineages present in a specimen, a predominant lineage can usually be assigned based on presence in at least 60% of the sequence reads, and nucleotide variants only included from the predominant lineage; and, if a predominant lineage cannot be assigned (~50/50 of each lineage), that sample excluded (for more detail, see Cullen et al., [38]). 

To address whether even finer genetic variation (i.e., SNPs) is associated with HPV16 carcinogenicity, we evaluated non-lineage-specific SNPs and the distribution of rare variants occurring within HPV16 lineages in a collection of 5,570 HPV16-infected case-control samples [37]. Thousands of unique HPV16 viral isolates were identified among women, suggesting that each may represent a distinct viral genome sequence possibly differing in carcinogenic potential. The controls had higher levels of rare sequence variants (particularly nonsynonymous and nonsense variants, i.e., amino acid changing) compared with cases across the genome and in specific regions. Interestingly, focusing on non-silent variation, *E7* was more variable in the controls compared to the cases, and we confirmed that *E7* showed extremely low variability in ~1700 cervical cancers from around the world. The *E7* gene was significantly less variable than all other gene regions in the cancers, including *E*6. This highlights that genetic conservation of *E7* (but not *E6*) is critical for HPV16 carcinogenesis. These rare nucleotide variants in *E7* frequently occurred in DNA motifs that are associated with the antiviral activity of human APOBEC3 (apolipoprotein B mRNA-editing, enzyme-catalytic polypeptide-like 3) family of cytidine deaminases. Interestingly, the majority of cervical cancer cases have been shown to be enriched for somatic APOBEC mutation signatures, suggesting that APOBEC antiviral activity is also a major source of somatic mutations in cervical cancers [45,46]. 

As discussed by the panel, these findings suggest that genetic variation within specific regions of the genome may differentially allow the virus to be cleared by the host; alternatively, the phenotype of cancer may require a fixed set of nucleotide variants at the viral genome level. 

## 3. Summary/Next Steps: Molecular Mechanisms Underlying HPV Carcinogenesis 

The Workshop agenda outlined many key questions related to three broad topic areas for discussion (Table 1). The first two sessions addressed molecular mechanisms underlying HPV carcinogenesis at the type and variant lineage/sublineage level, respectively. Each attendee was asked to contribute to these discussions by providing their unique perspective based on their expertise, for example, in basic science, epidemiology and/or computational biology. The following sections present an overview of the major themes that emerged. 

### 3.1. Viral Molecules and Their Interactions with Host Cellular Machinery 

The HPV life cycle is tightly linked to the differentiation state of infected epithelial cells. HPV infects basal keratinocytes that are exposed as the result of micro-abrasions in the epithelial surface [47,48]. Viral genome replication occurs at low levels in the basal layer and, as infected cells undergo terminal differentiation in the upper layers of the epithelium, E6 and E7 drive genome amplification by promoting cell-cycle re-entry and proliferation of HPV-infected cells [49,50]. Functional differences in E6 and E7 are thought to determine differences in carcinogenicity between high-risk and low-risk HPV types. Further, differences in the regulation of host protein interactions have been observed across high-risk types, which may contribute to known differences in carcinogenicity [51,52]. In addition, most viral proteins are expressed from spliced RNA molecules that have a complex regulation. Correlated changes across the viral genome could account for large changes in infection outcome (clearance, persistence and progression), as described in epidemiologic studies. 

### 3.2. Tissue Tropism and Site of Infection

HPV infections can occur within specific sites of transitional epithelial cells (e.g., the squamocolumnar junction) with complex patterns of regulation that may render them more prone to viral transformation [48,53,54]. At glandular sites such as the endocervix and tonsils, the diagnosis and/or presence of precursor lesions compared to invasive cancers is much lower than the diagnosis of cervical squamous intraepithelial lesions to squamous cell cancers [55]. Whether this is due to the position of the lesions and impact on screening efficacy, morphologic features of the precursors, or is related to actual differences in HPV natural history and oncogenesis is not fully understood [56]. Since E6 and E7 may induce a stem cell-like state, the type of cell that becomes infected may contribute to disease outcome. More research is needed to understand whether patterns of viral gene expression and protein function are site-specific, and how they vary across different high-risk HPV types. Specific observations that need explanation include the relatively high prevalence of precancers (i.e., CIN3) that do not translate into similar rates of cancer, suggesting that precancer is a distinct endpoint that does not serve as a perfect surrogate for cancer risk. For example, the ratio of HPV31 to HPV16 is much higher for CIN3 than for cancer, suggesting that a higher proportion of CIN3s caused by HPV31 do not lead to cancer.

### 3.3. Regulation of HPV Transcription

High-risk HPV types have evolved regulatory strategies to tightly control viral gene expression during productive and quiescent infections. Because of its critical role in regulating gene expression at different stages of the HPV life cycle, mRNA splicing efficiency may contribute to carcinogenic potential [57]. Recent findings from The Cancer Genome Atlas (TCGA) suggest that in HPV16 there is a lower ratio of spliced E6 transcripts (coding for E7) to unspliced transcripts (coding for E6) compared with HPV18 [45]. Thus providing some of the evidence that mechanisms of carcinogenesis differ between these *Alphapapillomaviruses-9* and *Alphapapillomaviruses-7* genomes and probably relates to their genetic differences. Efficiency of splicing may also differ [57,58], since HPV mRNA splicing and polyadenylation are regulated by cis-acting HPV RNA elements and cellular RNA-binding proteins. Synonymous, nonsynonymous and non-coding sequence differences in binding motifs or RNA structures may induce subtle changes in splicing and/or polyadenylation efficiencies that could have significant effects on viral gene expression and thus, carcinogenicity. However, to date, over 1000 host RNA binding proteins have been identified [59] and their ability to recognize and bind to multiple sequence motifs makes it particularly challenging to predict differences in splicing and/or polyadenylation across types or lineages by sequencing alone. In addition to primary sequence variation, secondary structures may influence the efficiency of RNA binding and can be difficult to predict. One approach discussed at the meeting involves transfecting various HPV16 isolates differing in oncogenicity and measuring viral RNA molecules through RNA sequencing approaches (RNA-Seq or whole transcriptome sequencing).

In vitro studies have recently shown that HPV16 and related types express a fusion protein encoded by subregions of the *E1* and *E*2 ORFs (termed E8^E2), which limits viral transcription and replication through the virus life cycle in undifferentiated keratinocytes [60]. This may play an important role in keeping the expression of early viral proteins at low levels so as to evade immune detection. Whether this protein occurs in natural infections remains untested, but it is an additional region that should be evaluated for any genome variation that might influence the viral life cycle and pathogenesis. 

### 3.4. HPV Integration into Host Genomes

Integration of HPV DNA into the host genome occurs in the majority of cervical cancers, but not all [61,62]. Mechanisms by which HPV integrates into the host cell genome and promotes carcinogenesis are not well understood. Sites of integration tend to occur in regions of genomic instability [63,64,65], and have also been reported to occur in short regions of HPV and host genome sequence homology (i.e., “micro-homologies”) [66,67,68], suggesting a potential role for DNA repair processes in integrating the HPV and host cell genomes based on nucleotide sequence similarities [69]. The prevalence of HPV integration in cervical cancers has been shown to vary by type, with lower frequencies observed for HPV types 31 and 33, compared with HPV types 16, 18, and 45 [61,69]. As a finer distinction, not all HPV16-associated cancers have integrated HPV DNA, whereas HPV18 integration is present in almost all HPV18-associated cancers. Viral-cellular fusion transcripts have been detected in all HPV18-positive cancers, some occurring in previously identified hotspots, such as 8q24 [45]. Interestingly, integration events associated with HPV18 appear to be more common at 8q24.21 near the *MYC* oncogene compared with HPV16-associated cancers [69,70]. At the HPV variant level, a recent study characterizing integration events by the HPV16 D and A variant lineages suggested differences in variant-specific integration potential, potentially mediated by E6 [71]. More studies are needed to confirm these findings and determine if and how viral genetic variation might relate to integration. 

### 3.5. Viral–Host Interactions

In response to infection with HPV, humans can mount an adaptive immune response including the development of specific antibodies to the virion L1 coat protein. Antibody and/or human cytotoxic T-lymphocyte (CTL) epitopes have been predicted within the peptides encoded by all HPV16 ORFs: 100% of *E5*, *E6*, and *E7* residues; 65–83% of *E2*, *E4*, and *L1* residues; and only 7% of *E1* residues (Immune Epitope Database). *E6* and *E2* epitopes appear to be the most important for a CTL response [72]. 

Alternative approaches for identifying potentially important HPV epitopes are based on evolutionary methods to identify positive selection that might indicate a pressure for immune escape, and these have mainly detected codons in the *E5*, *E6*, *L1*, and *L2* ORFs [11,73]. Future research in this area will take advantage of increasingly available sequence data to detect regions undergoing positive selection within sublineages, helping to elucidate sublineage- and case/control-specific immune responses. 

Both genetic and environmental host factors play key roles in determining viral oncogenicity. Epidemiologically defined co-factors, such as smoking and use of hormonal contraceptives, also play a role—for example, smoking has been associated with an approximate two-fold increased risk of precancer and cancer [74]. One goal of future research should be to link both host factors/genetics and viral genetics to infection outcomes. The host human leukocyte antigen (HLA) allele repertoire in particular, which is crucial for cell-mediated immune responses, may be a critical factor in determining which HPV variants will clear, and which will persist and potentially evade the immune system. In fact, these host immune alleles show signals for an inherited risk of cervix precancer/cancer [75,76,77]. Furthermore, specific HLA class I alleles have been associated with the oncogenicity of specific HPV16 variants [78,79], which highlights the importance of the HLA type combined with the HPV16 variants for immune surveillance in cervical carcinogenesis. The development of cancer may include such steps as an HPV variant infecting a host, who has an insufficient HLA repertoire for clearing that particular variant. 

## 4. Synthesizing Current Knowledge and Moving Forward in the Era of NGS, Systems Biology, and Big Data

The final session covered a range of topics related to characterizing and defining HPV fitness, annotation of HPV genomes, and host-viral interactions. These topics have important implications for HPV genomics research and could serve as a model for other genetic systems. 

### 4.1. Defining HPV “Fitness”

Evolutionary fitness in biology is usually defined as reproductive success. The definition of how to define viral fitness in general, and HPV fitness in particular, remains unresolved and was not agreed upon by the workshop panel. An increase in viral replicative success may have conflicting proximal and ultimate outcomes for the host. For example, a particular viral genotype may replicate to high viral load in a particular cell, but the outcome may be to increase the likelihood of an immune response, thereby drastically decreasing the actual fitness of the virus. Surprisingly, much of the feedback from the panel was that a consensus definition for fitness might not be useful for describing carcinogenic features of different HPV types and variants. The intellectual divide resided in whether viral evolution, niche adaptation, and fitness represent the key drivers of carcinogenesis, although carcinogenesis does not support viral replication. Some attendees suggested a more direct paradigm using viral outcomes, such as causing cancer or not, as the “viral” phenotype (Table 2). Others suggested defining fitness as viral prevalence in the infected population, i.e., the outcome of incidence and persistence. In addition, the steps to cancer can be considered either as independent outcomes (e.g., persistence, precancer), or as a sequential set of steps that could be studied using functional assays. 

Given that the most prevalent anogenital HPV type (HPV16) is also the most carcinogenic, future research should consider the relationship between viral prevalence and oncogenicity. It appears that viral traits that improve reproductive success also tend to initiate processes that predispose host cells to cancer. In either case, the ability to induce cancer is neither necessarily, nor inextricably, linked to HPV’s ability to successfully propagate in populations, such that oncogenicity may be considered an unfortunate byproduct that is not itself under selection. Adaptation to a specific cellular environment may define features of the HPV genome that induce cancer as “collateral damage” rather than a selective trait, since cancer does not support the production of infectious virus.

### 4.2. HPV Genome Annotation and Other New Emerging Data Concepts

The importance of genome annotation is critical for evaluating the impact of sequence variations across viral variants and between viral types. In fact, since the variation between viral types is so large (approximately 30%), unequivocal alignment and position assignments are not currently feasible. Therefore, annotation of functions could serve as a common database to connect different features of disparate genomes. The way forward was not defined, but a bioinformatic approach is a promising area that could build upon work done on the annotation of various mammalian genomes that face similar challenges.

## 5. Conclusions

The field of HPV genomics is undergoing a major paradigm shift from thinking of an HPV type infection as an evolutionarily static entity to thinking of thousands of unique viral genomes with differences in carcinogenic potential. Findings from recent large epidemiologic studies defining the association of HPV variant lineages/sublineages/SNPs with cervical cancer risk have led to new discoveries that call for HPV natural history and carcinogenesis to be re-visited. These findings also merit additional experimental studies using tools developed in the “omics” era. These novel discoveries underscore the importance of designing relevant comparisons to help sort out the differences in viral genetic features of carcinogenesis at the biochemical and mechanistic level. For example, across HPV genotypes, a large number of nucleotide differences may reveal more broad associations between HPV type and processes such as viral–host interactions, tissue tropism at the cellular level, splicing, and protein translation. In contrast, variant lineage/sublineage studies within a particular HPV type will allow for the identification of individual variants, or small groups of variant sites (haplotypes), related to differences in carcinogenicity. Integrating epidemiologic findings with functional studies may transform our basic understanding of HPV-associated carcinogenesis and may eventually elucidate the genetic basis defining what makes some HPVs, especially HPV16, such powerful carcinogens. 

HPV carcinogenesis is a multifactorial complex process that involves a confluence of viral and host factors. However, compared with the complexities associated with studying the human genome, the genetic basis of HPV carcinogenicity in an 8000 bp genome is a more tractable problem that deserves immediate attention.

## Figures and Tables

**Table 1:** Topics and specific questions addressed at the National Cancer Institute (NCI) HPV Workshop.

Topic Area	Questions
HPV studies at the species and/or type level and risk of cancer	What features of the biology and/or biochemistry of HPV16 make it so uniquely carcinogenic?
	What features of HPV16 biology, or interaction with the host cells, enable it to have a wider tissue tropism and disease association?
	What are the experimental approaches to investigate this? This should include comparisons of closely related HPV types (i.e., HPV16, 31, and 35) with large differences in carcinogenesis Comparison of appropriate type(s) to HPV16/18 biology/biochemistry (HPV6/11 are probably not a good choice because they are evolutionarily distant)
Studies of HPV variant lineages within a type to elucidate differences and risk of cancer	Are there functional differences between HPV16 A1 vs. D sublineage viruses that could help explain their pathological differences in cancer risk? How do we mechanistically explain the genetic variants associated with glandular lesions for the HPV16 A4, D2, D3 sublineages compared to A1 and A2?
	Why are specific HPV16 sublineages (i.e., A4, D2, D3), and HPV18 and HPV45 prone to adenocarcinoma?
	What are the next steps after the SNP/gene-based epidemiological approach using case-control datasets? What cell based and/or biochemical experiments could be used to identify the mechanisms of different genetic associations?
Synthesizing current knowledge and moving forward in the era of NGS, systems biology and large data sets	How do we define the characteristics of HPV fitness?
	How do we annotate HPV genomes to be able to capture common functional motifs with disparate genomes in large datasets? (e.g., it’s hard to align a large number of genome sequences of HPV16 with HPV31, HPV52, etc) How do we go beyond the annotations in NCBI and PaVE?
	How do we incorporate information on viral suppression/invisibility to the host immune system?
	Where does epigenetics of the viral genome fit into the discussion of dissecting viral genome differences?
	How to best approach viral—host interactions?

**Table 2:** Distinct steps in the pathway from HPV infection to carcinogenesis.

Functional Step	Relevant Features
1. Infection	Cell receptor(s) for entry
	Tissue tropism
2. Persistence	Continued productive infection
	Persistence without productive infection
	Cellular immunity
	Latency
	Early, inapparent transformation
3. Transformation	Increased E6/E7 expression
	Chromosomal instability
	Somatic mutations
	Viral integration
4. Invasion	Increasing somatic mutations
	Integration, disruption, and partial loss of viral genome
	Epithelial-stromal interactions

## Supplementary Materials

The following are available online at http://www.mdpi.com/1999-4915/10/2/80/s1, Table S1: Full list of workshop attendees.
